# Annotations

**Published:** 1893-04-15

**Authors:** 


### Annotations.

**Doctors and Death.** The deaths of several well-known medical practitioners lately at ages but little beyond the middle period of life, have given a shock to a good many of their professional brethren. And no wonder! One of the first things a doctor naturally aims at is the preservation of his own health and life. If he cannot keep himself alive and well, the prospect is certainly not encouraging for his patients. It is commonly believed that doctors are a short-lived class. Is the popular belief well founded? It is, unhappily, only too well founded. In his "Vital Statistics," Dr. Arthur Newsholme informs us that of every 1,000 clergymen between the ages of 45 and 65, only 15·93 die annually. But of every 1,000 doctors between the ages of 45 and 65, no fewer than 28·03 die every year. That is to say, the mortality of medical men is almost double that of clergymen. And the rate is increasing. Twenty years ago it was only 24 instead of 28. These are facts to give us pause. They seem to throw an ugly doubt upon the competency of our professional skill. It may perhaps be argued that clergymen are financially more prosperous than medical men; but that would be quite fallacious; because Dr. Newsholme includes in his clerical class the ministers of all the Nonconforming denominations; and class for class, there is no doubt that medical men are much better off than the body of clergymen as thus constituted. What is the reason then for this portentous death-roll? It is to be found in the arduous nature of the doctor's daily work. But then the doctor knows the arduousness of his work, and if he had faith in physiology he would counteract the effect of that arduousness by more holidays, and by combining with his fellow doctors, to diminish the killing strain. This is a matter which is generally talked about and then dropped. The writer intends, however, to take some practical steps for amelioration, and within a reasonable time hopes to be in a condition to ask for the co-operation of his hardly-used professional brethren.

**A Lesson for Mr. Stead.** The man with a club in his hand appears to be a much more effectual fighter than the more delicate-looking man of science. Nevertheless it is the men of science who are conquering the world; and the wielders of clubs have been the first to go down before them. In medicine the quack and the pretender use the club of the savage. Self-confidence, bluster, absolute certainty of cure are their weapons of war. They pile up testimonials by the thousand, and carry the citadel of faith and fees with a rush. But the real healers and wonder-workers in medicine, the men who have raised physic out of the deep mire of ignorance and crude guesswork, have none of these methods. Their confidence is in the "still small voice" of truth and reality. Most striking, in this connection, are the words of a distinguished army surgeon in the *Practitioner* for April. Surgeon-Lieutenant-Colonel A. Crombie reports in that periodical a series of twenty-two cases of pneumonia treated by him after a new method, and treated with singular success. Dr. Crombie used calcium chloride for pneumonia in his twenty-two cases, and the results of his treatment were, that whereas the ordinary mortality in such cases is from twenty to forty per cent., the mortality among his patients was under five per cent. With such results a charlatan would astonish the world; and, what is so deplorable, the world would consent to be astonished, and to part with its money with reckless profusion. What does Dr. Crombie say? He says to his professional brethren: "I would not come before you with this record and ask you to give a trial to a new treatment if my cases had been less numerous than they are, or had exhibited less uniformity in what appeared to be their response to the treatment. It may be that I have been the sport of circumstances, and have merely been treating a series of mild or fortunate cases. . . . . But I have placed my cases before you, leaving you to decide whether they are sufficiently encouraging to justify you in giving the new treatment an adequate trial." Now there is moral nobility here as well as scientific simplicity. Dr. Crombie is not conscious of any moral nobility. He knows that he is simply doing as any other right-minded medical man would do. But medical work, or any other work, carried on in this spirit of truthful candour is a moral leaven working in society which, in due time, may leaven the whole lump with honesty and childlike simplicity. Mr. Stead is a champion of moral purity. Has he the intellectual capacity to see the gradual but inevitable moral effect upon the social organism of such methods of work as have just been described.

**Stands Edinburgh for Untrained Nurses?** Practical people cannot but be surprised at the resolution passed the other day at Edinburgh, which indicated that certain not unknown medical men in that city are of opinion that a young woman may be registered as a competent and qualified nurse after only one year's training. If such a resolution should be accepted by the Royal British Nurses' Association in London, to whom it was referred by the meeting, it will probably give the *coup de grace* to the pretensions of that association to be a promoter of nurse education and training, and a defender of the interests of the trained against the self-assertion of the half-trained or the untrained. London has always insisted upon a high standard of education and acquirement for medical men; and, consistently with its past, it now insists upon a high standard for hospital-trained nurses. Is there any experienced person anywhere who will affirm that three years is too long a period of training for the average young woman who devotes herself to nursing? But it is the average young woman we must legislate for if our legislation is to be of a statesmanlike and permanent character. One year of training is very little better than no training at all. A woman hardly begins to appreciate her own ignorance or the nature and difficulties of hospital work in that short time. One of the chief contentions which the nurse training schools in London and elsewhere has always had with the B.N.A. is that, in order to swell its own ranks, it sweeps the trained, the half-trained, and the untrained into one common net, and thus swamps the competent, the experienced, and the worthy by the mass of the ignorant and the unproved. We do not venture to address any remonstrance to the leaders of the B.N.A. on this point. They must be left to their own devices. But we do very urgently call the attention of the leaders of the nurse-training schools to this extraordinary attempt to "hark back" to the "Gamp" period, and to undo all the noble educational work which Miss Nightingale and others have effected by the strenuous and admirable labours of a life-time. We are glad to see that neither the University nor the Royal Infirmary of Edinburgh appear to have taken any official part in the proceedings. We sympathise with the University which, like London, has always affirmed the necessity for a high standard of medical education. There are other medical institutions in the Scottish capital which have a very different history; and the names of some of their managers were conspicuous as supporters of this fatal attempt to drag down the standard of nurse-training and education. Stands modern Edinburgh for ignorance and half-culture? It is enough to make the ghosts of Jeffrey, Christopher North, Christison, and the other great ones of the past, pull down the Scott monument and raze the College of Surgeons to the ground

